# A structured literature review of the burden of illness and unmet needs in patients with rheumatoid arthritis: a current perspective

**DOI:** 10.1007/s00296-015-3415-x

**Published:** 2016-01-08

**Authors:** Peter C. Taylor, Adam Moore, Radu Vasilescu, Jose Alvir, Miriam Tarallo

**Affiliations:** Rheumatology and Musculoskeletal Sciences, Kennedy Institute of Rheumatology, University of Oxford, Oxford, UK; Endpoint Development and Outcomes Assessment, Adelphi Values, Cheshire, UK; Pfizer Regional Medical Europe, Brussels, Belgium; Global Innovative Pharma Business, Pfizer Inc, New York, NY USA; Global Health & Value-GIP, Pfizer, Rome, Italy; ICE Creates Ltd, Birkenhead, UK

**Keywords:** Cost, Fatigue, Mental functioning, Pain, Physical functioning, Rheumatoid arthritis

## Abstract

While rheumatologists often focus on treatment targets, for many patients with rheumatoid arthritis (RA), control over pain and fatigue, as well as sustaining physical function and quality of life (QoL), is of primary importance. This literature review aimed at examining patients’ and physicians’ treatment aspirations, and identifying the unmet needs for patients with RA receiving ongoing treatment. Searches were performed using MEDLINE, Embase, PsycINFO, and Econlit literature databases for articles published from 2004 to 2014 in the English language. Published literature was screened to identify articles reporting the unmet needs in RA. We found that, despite the wide range of available treatments, RA continues to pose a substantial humanistic and economic burden on patients, and there are still unmet needs across key domains such as pain, physical function, mental function, and fatigue. These findings suggest that there is a need for further treatment advances in RA that address these domains of contemporary unmet need.

## Introduction

Rheumatoid arthritis (RA) is a chronic inflammatory disease characterized by pain and stiffness of affected joints, with an estimated global prevalence of 0.3–1.0 % [1]. Fatigue, joint inflammation, and deformities are key complications of RA leading to impaired physical functioning, work productivity, and activities of daily living, which can also compromise overall emotional well-being [2]. Suboptimal treatment can exacerbate this decline, causing a considerable burden to patients and a substantial strain on global healthcare resources [3, 4].

In the contemporary treatment paradigm, methotrexate (MTX)—a conventional synthetic disease-modifying antirheumatic drug (csDMARD)—is the recommended first-line treatment for patients with RA, often administered in combination with other csDMARDs. In patients with an inadequate response to first-line csDMARDs, MTX is generally used in combination with a concomitant biologic DMARD (bDMARD) such as a tumor necrosis factor (TNF) inhibitor [5, 6].

Although the achievable outcomes for RA continue to evolve and improve, not all patients are able to attain the desirable treatment goal of remission or, failing that, of low disease activity (LDA). While the emphasis of rheumatology care has been on attainment of these treatment targets, for many patients, particularly those in whom these targets are not achieved, control over symptoms such as pain and fatigue, as well as maintaining physical function and quality of life (QoL), is of primary importance. Therefore, mitigating the negative impact of the disease on patients’ lives and QoL, reducing or halting disability, and achieving clinical remission continue to be a focus of research [7–10].

The primary objectives of this literature review were to identify and summarize the unmet needs of patients with RA despite receiving ongoing treatment with csDMARDs with or without concomitant biologic therapies.

The secondary objectives were to investigate the humanistic and economic burden of RA, the potential discordance between patients’ treatment goals and perceptions of well-being, and physicians’ therapeutic targets based primarily upon disease activity assessments.

## Patients and methods

### Assessment of unmet medical needs in RA: literature search and review methodology

Searches were conducted in March 2014 using MEDLINE, Embase, PsycINFO, and Econlit literature databases and limited to human studies published in English from January 2004 to March 2014. Published literature was screened using search terms combined with subsearches to identify articles reporting the burden of RA in patients receiving ongoing treatment (Table 1).

**Table 1:** Core search terms and subsearches

Evidence requirement	Search terms
Condition (required to be in title)	Rheumatoid arthritis **OR** RA
AND	
Treatment	Biologic$ **OR** injection$ **OR** injectable **OR** oral$ **OR** pill$ **OR** tablet$ **OR** DMARD **OR** disease-modifying antirheumatic drug$
Subsearch 1: Humanistic burden	Quality of life **OR** QoL **OR** health-related quality of life **OR** HRQL **OR** HRQoL **OR** activities of daily living **OR** hobbies **OR** physical functioning **OR** social impact **OR** emotional impact **OR** social interaction **OR** isolation **OR** physical ability **OR** mental function **OR** psychological function **OR** work disability **OR** sexual function **OR** pain **OR** stiffness **OR** stiff **OR** loss of strength **OR** loss of movement **OR** fatigue **OR** joint function **OR** swollen joint$ **OR** swelling **OR** patient preference **OR** needle phobia **OR** adverse events **OR** side effects **OR** adherence **OR** treatment discontinuation **OR** treatment burden **OR** patient impact **OR** burden of illness **OR** treatment convenience **OR** treatment administration **OR** treatment preparation **OR** dosing schedule **OR** treatment frequency
Subsearch 2 Economic burden	Cost of illness **OR** healthcare cost **OR** economic burden **OR** economic impact **OR** resource use **OR** hospitalization **OR** productivity **OR** expenditure **OR** cost utility **OR** absenteeism **OR** cost **OR** economic

*DMARD* disease-modifying antirheumatic drug, *HRQoL* health-related quality of life, *QoL* quality of life, *RA* rheumatoid arthritis

Two levels of core search terms were included: One related to the condition of study, and the second used treatment-related search terms. Articles that included terms from both of these levels were identified by two researchers. Search terms related to treatments were included as a key objective of the search. Following the exclusion of duplicate articles across the humanistic and economic burden subsearches, articles were identified for inclusion. Titles of articles were screened to exclude any articles that could be deemed irrelevant; articles and abstracts were screened and excluded if relevant terms were included as background, as an implication in the discussion, or were lacking in data. All conference abstracts were excluded from this review.

Key outcome measures included aspects of life important to patients such as pain, physical functioning, mental functioning, fatigue, social functioning, sexual functioning, and treatment-related issues, as well as impact on work and economic burden. Minimal clinically important difference (MCID) values were utilized when available to assess the magnitude of changes over time. In addition, patient acceptable symptom state (PASS) values were used when available to determine whether the observed values would be acceptable to patients with RA (Table 2).

**Table 2:** Established MCID and PASS values across a range of commonly utilized outcome measures

Score	MCID (point change)	Supporting reference	PASS^a^
HAQ	–0.2	Wells et al. [16]	1.0
SF-36 PCS	2.5	Strand and Singh [56]	N/A
Physical function	5.0		50.0
Role-physical	5.0		N/A
Bodily pain	5.0		41.0
General health	5.0		47.0
SF-36 MCS	2.5		N/A
Role-emotional	5.0		N/A
Vitality	5.0		40.0
Social function	5.0		75.0
Mental health	5.0		68.0
Pain (VAS)	–11.8	Pope et al. [57]	34.0
Fatigue (VAS)	–10.0	Wells et al. [58]	50.0

*HAQ* health assessment questionnaire, *MCID* minimal clinically important difference, *MCS* mental component score, *N/A* not applicable, *PASS* patient acceptable symptom state, *PCS* physical component summary, *SF*-*36* medical outcomes short form-36, *VAS* visual analog scale

^a^The PASS are all reported in a single article [79]

## Results

The search identified 3212 unique articles; 1688 were excluded as it was clear from their title that they were irrelevant to the goals of the study, or that RA was not the focus of the article. Of the remaining 1524 articles, 1447 were removed at the abstract screening stage. In total, the search identified 77 key publications that reported on the humanistic (68 articles) and economic burden (9 articles) of RA.

### Pain

In total, 13 articles (comprising 14 cohorts) were identified that discussed the impact of pain in patients with RA, in line with the objectives of this review. Four cohorts fulfilled PASS after intervention treatment/observational period, while 4 cohorts (from 15 with available data) fulfilled PASS based on a cross-sectional design. 7/14 cohorts with MCID available fulfilled the required threshold. Overall, the literature suggests that while biologics in combination with MTX alleviate pain, many patients with RA continue to experience unacceptable levels of pain (Table 3). Data from clinical trials demonstrated that MTX in combination with a biologic resulted in greater reduction in pain compared with MTX monotherapy [11].

**Table 3:** Summary of pain, physical functioning, and SF-36 mental component summary scores observed across the reviewed studies

Reference	Treatment	Mean disease duration (years)	Study type	Study duration*	Pain		HAQ^†^				SF-36 mental component summary	
					MCID^a^	PASS^b^	Baseline	End of study	MCID	PASS	MCID	PASS
*Biologics*												
[11]	PL	10.4	Clinical trial	N/S	No	No	N/A	N/A	N/A	N/A	N/A	N/A
	CZP	8.7			Yes	No	N/A	N/A	N/A	N/A	N/A	N/A
	CZP 200 mg	N/S			N/A	N/A	1.6	1.0^††^	70 %^e^	Yes	Yes	N/A
	CZP 400 mg	N/S			N/A	N/A	1.7	1.0^††^	70 %^e^	Yes	Yes	N/A
[21]	ETN + MTX	6.0	Observational	N/S	Yes	Yes	1.2	0.6	Yes	Yes	N/A	N/A
[19]	ETN	12.5	Observational	N/S	N/A	N/A	1.7	1.3	yes	no	No	N/A
	Control	12.3		N/S	N/A	N/A	1.8	1.6	no	no	No	N/A
[59]	bDMARDs + biologics: females	N/S	Observational	8***	No	No	0.9	0.9	No	Yes	N/A	N/A
	DMARDs + biologics: males	N/S		N/S	Yes	No	0.8	0.4	Yes	Yes^d^	N/A	N/A
[60]	Continuous biologic use	20.3	Observational	N/S	No	No	1.2	1.2	No	No	N/A	N/A
	Discontinued biologic use	20.7			No	No	1.2	1.2	No	No	N/A	N/A
	No biologics	20.9			No	No	0.9	0.9	No	Yes	N/A	N/A
[61]	All patients	14.0	Observational	N/S	No	No	N/A	N/A	N/A	N/A	N/A	N/A
	Started with biologics	N/S			Yes	No	N/A	N/A	N/A	N/A	N/A	N/A
	Started with MTX	N/S			No	No	N/A	N/A	N/A	N/A	N/A	N/A
[62]	INF, ETN + ADA	9.4	Observational	N/A	N/A	No	N/A	N/A	N/A	N/A	N/A	N/A
	TNF-α inhibitors: female	9.3				N/A	1.1	N/A	N/A	No	N/A	N/A
	TNF-α inhibitors: male	9.6				N/A	0.9			Yes	N/A	N/A
[66]	Total	12.8	Observational	N/S	N/A	N/A	1.1	N/A	N/A	No	N/A	N/A
	TNF-α inhibitors users	12.5				No	0.9			Yes	N/A	N/A
	TNF-α inhibitors naive	14.1				Yes	0.7			Yes	N/A	N/A
[20]	ETN + MTX	6.5	Clinical trial	16	N/A	N/A	1.4	0.7	Yes	Yes	Yes	N/A
	DMARD + MTX	6.9					1.4	0.9	Yes	Yes	Yes	N/A
[15]	ABA + DMARD	12.2	Clinical trial	6**	N/A	N/A	1.8	1.3	Yes	No	MCS, V, SF, RE	V
	PL + DMARD	11.4					1.8	1.7	No	No	MCS only	None
[67]	ABA	8.0	Clinical trial	N/A	N/A	N/A	1.5	N/A	N/A	No	N/A	N/A
	INF						1.5			No	N/A	N/A
	RTX						1.7			No	N/A	N/A
[71]	TNF-α inhibitors	13.0	Observational	5***	N/A	N/A	1.6	1.2	Yes	No	Yes	N/A
[18]	Biologics	12.7	Observational	6**	N/A	N/A	1.2	1.1	No	No	N/A	N/A
*Syk inhibitor*												
[23]	Fostamatinib 100 mg + MTX	8.4	Clinical trial	N/S	Yes	Yes	1.5	1.0	Yes	Yes	N/A	N/A
	Fostamatinib 150 mg + MTX	9.7			Yes	Yes	1.5	0.9	Yes	Yes	N/A	V only
	PL + MTX	9.5			Yes	No	1.5	1.2	No	No	N/A	N/A
*DMARDs*												
[12]	DMARD	N/S	Cross-sectional	N/S	N/A	No	N/A	N/A	N/A	N/A	N/A	MH only
[61]	DMARD + biologics 1997 cohort	15.0	Observational	N/S	N/A	No	1.2	N/A	N/A	No	N/A	V/MH
	2002 cohort	17.0					N/A	N/A	N/A	N/A	N/A	N/A
[63]	2005 cohort	14.0	Observational				0.9	N/A	N/A	Yes	No	V, MH
[64]	DMARD High GDP, working	11.0	Cross-sectional	N/S	N/A	Yes	N/A	N/A	N/A	N/A	N/A	N/A
	High GDP, not working					No		N/A	N/A	N/A	N/A	N/A
	High GDP, all patients					Yes		N/A	N/A	N/A	N/A	N/A
	Low GDP, working					No		N/A	N/A	N/A	N/A	N/A
	Low GDP, not working							N/A	N/A	N/A	N/A	N/A
	Low GDP, all patients							N/A	N/A	N/A	N/A	N/A
[65]	DMARD 1994 cohort	10.8	Observational	N/S	N/A	No	N/A	N/A	N/A	N/A	N/A	MH only
	2001 cohort	13.8				N/A	N/A	N/A	N/A	N/A	N/A	V, MH
	2004 cohort	13.6				Yes	N/A	N/A	N/A	N/A	N/A	N/A
[68]	DMARD	9.0	Observational	N/A	N/A	N/A	0.5^††^	N/A	N/A	Yes	N/A	N/A
[32]	DMARD + biologics	N/S	Observational	36**	N/A	N/A	1.9	0.8	Yes	Yes	SF, RE	V and SF
[22]	DMARDs	≤2	Observational	12**	N/A	N/A	1.7	1.1	64 %^e^	No	Yes	N/A
[69]	DMARDs	6.6	Observational	12**	N/A	N/A	1.1	N/S	N/A	No	N/A	N/A
[37]	DMARDs	N/S	Observational	5***	N/A	N/A	0.9	0.8	No	Yes	N/A	N/A
[72]	DMARDs	7.0**	Observational	10***	N/A	N/A	0.7	0.4	Yes	Yes^c^	N/A	N/A
[73]	DMARDs	11.9	Observational	27**	N/A	N/A	0.9^††^	0.5^††^	Yes	Yes^c^	N/A	N/A
[74]	No treatment + DMARDs + biologics	9.7	Cross-sectional	N/A	N/A	N/A	1.3	N/A	N/A	No	N/A	N/A
[2]	DMARD + biologics	9.4	Cross-sectional	N/A	N/A	N/A	1.0	N/A	N/A	Yes	N/A	N/A
[75]	DMARDs	12.5	Cross-sectional	N/A	N/A	N/A	1.3	N/A	N/A	No	N/A	N/A
[24]	DMARDs + biologics	12.5	Cross-sectional	N/A	N/A	N/A	1.6	N/A	N/A	No	N/A	V only
[76]	DMARDs + biologics	10.6	Cross-sectional	N/A	N/A	N/A	1.4	N/A	N/A	No	N/A	V only
[77]	DMARDs: males	5.9	Cross-sectional	N/S	N/A	N/A	N/A	N/A	N/A	N/A	N/A	MH only
	DMARDs: females	4.8		N/S	N/A	N/A	N/A	N/A	N/A	N/A	N/A	V, MH
*Corticosteroids*												
[70]	Corticosteroids	5.0	Observational	9**	N/A	N/A	1.4	1.0	Yes	Yes	N/A	N/A

*ABA* abatacept, *ADA* adalimumab, *CZP* certolizumab pegol, *DMARD* disease-modifying antirheumatic drug, *ETN* etanercept, *GDP* gross domestic product, *HAQ* health assessment questionnaire, *INF* infliximab, *MCID* minimum clinically important difference, *MCS* mental component score, *MH* mental health, *MTX* methotrexate; *N/A* not applicable, *N/S* not stated, *PASS* patient acceptable symptom state, *PL* placebo, *RE* role-emotional, *RTX* rituximab, *SF* social function, *SyK* spleen tyrosine kinase, *TNF* tumor necrosis factor, *V* vitality, *VAS* visual analog scale

* Data are expressed in weeks unless stated otherwise: ** months; *** years

^†^Data are mean, unless stated otherwise: ^††^median values

^a^Threshold value –11.8

^b^34 of 100 on 0–100 VAS

^c^Minimal residual activity achieved (based on a value of ≤ 0.5 [78], cross-sectional data)

^d^Minimal residual activity achieved (based on a value of ≤ 0.5 [78], clinical trial data)

^e^Data are proportion of patients achieving MCID, where stated

One study confirmed that although treatment with a biologic in patients produced clinically meaningful improvements in pain, scores remained below the PASS threshold (Table 3) [11]. In addition, patients with RA continue to experience moderate pain, despite ongoing treatment with DMARDs [12].

Interestingly, patients’ global assessment of disease accounted for 32.8 % of the variation in pain intensity and 10.7 % of the variation in morning stiffness; these outcomes were considered more important to patients than radiographic or clinical outcomes, such as the number of tender and swollen joints [13]. Overall, the current literature suggests that pain persists at an unacceptable level in patients with RA.

### Physical functioning

In line with the objectives of this review, 27 articles (comprising 29 cohorts) on physical functioning were identified. Seventeen cohorts fulfilled PASS after intervention treatment/observational period, while 13 cohorts (from 29 with available data) fulfilled PASS based on a cross-sectional design. 20/29 cohorts with MCID available fulfilled the required threshold. Overall, physical functioning outcomes persist at an unsatisfactory level in patients with RA, particularly in those who do not achieve MCID or PASS thresholds despite ongoing treatment (Table 3). Remaining independent and carrying out activities of daily living are paramount to patients with RA; as such, improved mobility and mitigating pain and fatigue have been identified as critical treatment goals [14].

Evidence in the literature suggests that mild to moderate disability (mean health assessment questionnaire [HAQ] score of 1.2–1.8 at baseline) is above the threshold that patients would consider acceptable [15, 16]. Clinical studies showed that continuing patients on csDMARDs, when they may benefit from treatment with biologics, failed to result in improved physical functioning [17], highlighting the advantages of switching to intensive treatment strategies in patients with an inadequate response to csDMARDs.

Consistent with findings from clinical trials, data from observational studies suggested that physical functioning scores, captured using patient-reported outcomes (PROs) such as the HAQ and the medical outcomes short form-36 (SF-36), failed to reach PASS thresholds and seldom reached clinical targets for minimal residual disease activity. These shortcomings were observed in patients receiving csDMARDs and biologics, suggesting that treatment goals—from patients’ and physicians’ perspectives—are rarely met with existing therapies.

Moreover, current evidence suggested that available therapies often fail to improve HAQ scores by clinically important margins, with patients frequently experiencing an unacceptable level of physical disability despite ongoing treatment [18, 19]. Furthermore, approximately 47 % of patients failed to achieve HAQ levels indicative of minimal residual disease activity (a secondary goal of treatment for patients unlikely to achieve remission) [20, 21].

There was no conclusive evidence in the reviewed literature to determine the effect of available treatments on morning stiffness in patients with RA. Overall, physical functioning continues to pose a problem for many patients with RA, despite ongoing treatment.

### Mental functioning

In line with the objectives of this review, 16 articles assessed mental functioning in patients with RA, using the four domains that comprise the mental component score (MCS) or the mental health subdomain of the SF-36. In general, suboptimal mental health persists in a substantial proportion of patients with RA (Table 3). Studies showed that approximately 48–92 % of patients who remained on MTX—despite meeting eligibility criteria for treatment with biologics—did not meet MCID thresholds [17]. Furthermore, 35–66 % of patients failed to meet MCID thresholds across six clinical trials of biologic treatments [17]. A study in a cohort of South African patients with early RA concluded that only 43 % of previously DMARD-naïve patients individually met the MCID threshold, and 66 % of patients had suboptimal mental health (SF-36 MCS < 66.6) after 12 months of DMARD therapy [22].

There were no studies that used disease-specific measures of mental health. Taken together, these findings suggest that there is an unmet mental health need, and failure to intensify treatment may result in mental health problems persisting in many patients with RA.

### Fatigue

Unacceptable levels of fatigue persist in a substantial proportion of patients with RA, despite the introduction of intensive treatments. Data from clinical trials demonstrated that biologics, in combination with MTX, often fail to produce meaningful improvements in fatigue and that patients with an inadequate response to MTX continue to experience substantial distress due to fatigue [20, 23]. Furthermore, high multidimensional assessment of fatigue scores was strongly correlated with disease activity, suggesting that fatigue is severer for patients with moderate to severe disease activity, compared with those with LDA [24].

Overall, the current literature suggests that fatigue continues to have a considerable negative impact on more than half of patients with RA [9] and is a major determinant of QoL [25–27]. The absence of fatigue, although rarely achieved, has previously been defined as a key component of one of the more stringent definitions of remission in RA [28]. Moreover, research has confirmed that 40–80 % of patients with RA believe that reducing fatigue should be a key treatment aim, although fatigue-related endpoints were rarely reported in clinical trials [27, 29, 30].

### Social functioning

The impact of RA on social functioning was not the primary focus for any of the reviewed articles. As such, limited evidence was available regarding the therapeutic potential of available treatments on social functioning in patients with RA. However, one study concluded that a negative impact on relationships with friends and family was reported by approximately one-fifth of patients with RA [31].

PASS values for social functioning were met in 1 of 10 studies and were achieved only in a subpopulation of the overall sample who had been receiving MTX at the start of the study period [32]. Of note, the PASS value for social functioning for patients with RA is much higher than that observed for other subdomains of the SF-36, highlighting the importance of maintaining high levels of social functioning in patients with RA. Overall, these data demonstrate that acceptable levels of social functioning may not be achievable for patients with RA, despite ongoing treatment.

### Sexual functioning

In patients with RA, sexual disability can manifest due to several factors, including joint pain and fatigue, difficulty in assuming certain positions when hip or knee movements are restricted, and diminished sexual desire [33].

In a 6-month observational study of sexual activity and sexual dysfunction in patients with RA receiving treatment with biologics or DMARDs, 53.8 % of men and 45.7 % of women experienced some form of sexual dysfunction in response to a multidimensional patient-reported outcome measures questionnaire [34]. One survey revealed 22 % of biologic-experienced and 16 % of biologic-naïve patients (*P* ≤ 0.05) experienced problems with sexual function [31]. However, these data were collected from one question asked as part of a telephone survey, rather than as part of a disease-specific PRO, and should therefore be interpreted with caution.

Although problems with sexual functioning continue to adversely affect patients with RA, data in the current literature are sparse, highlighting the unmet needs of patients with RA.

### The role of patients in management decisions regarding therapy

Perceived control over RA may be a key component in determining patients’ wishes to maintain or switch treatments. However, based on the reviewed literature, it was not possible to accurately ascertain how patients gauged control of RA.

There was evidence to suggest that patients’ fear of side effects was a key barrier to switching treatment and that non-adherence was often attributed to side effects associated with their current medication [35]. Of note, a post-marketing surveillance study concluded that patient-reported self-administration of medication led to feelings of independence (89.1 %) and improved QoL (83.6 %), and may be desirable for many patients with RA [36]. Based on the current literature, further research is warranted to understand patients’ experiences of RA treatment.

## Impact on work

The physical and mental effects of RA continue to be a challenge for patients with RA and often result in a substantial negative impact on patients’ ability to work. Despite this, efforts to reduce the negative work-related impacts of RA are not recognized treatment goals of existing therapies.

Historically, an estimated one-third of patients with RA terminate employment prematurely, and 5 years after diagnosis, 30–40 % of patients experience work disability [37–40]. Relationships between work disability and PROs that are of clinical importance such as HAQ, modified Health Assessment Questionnaire (mHAQ), and pain scores showed that increased severity of pain and physical disability were associated with greater work disability [41–43].

There was evidence in the literature that intensive treatment strategies with a combination of DMARDs may play a crucial role in reducing the adverse work-related impacts of RA [41, 44]. Reducing work disability in patients with RA is crucial as engaging in paid work has a positive effect on health-related quality of life (HRQoL) [12]. Novel treatments that adequately address pain and physical functioning, mitigating the negative work-related effects of RA, are eagerly awaited.

### Economic burden as an unmet need in RA at the societal level

RA is associated with a large economic burden to individual patients, their families, and to society, with an estimated total annual economic burden of €45.3 billion in Europe and €41.6 billion in the USA [45].

Direct costs associated with RA include medications, hospitalizations, clinic visits, laboratory monitoring imaging, toxicity, and medical assist devices. Indirect costs, such as loss of earnings, caregiver productivity, and intangible costs arising from pain, depression and anxiety, and suboptimal QoL also contribute to the economic burden of RA [46]. Of note, overall costs were greater for those treated with multiple versus single TNF-α inhibitors: ($8340 vs. $7058), as were RA-related healthcare costs ($15,048 vs. $13,312) and total healthcare costs ($26,679 vs. $21,831) [47]. Studies of indirect costs generally focus on absenteeism associated with the disease, and there was limited research on presenteeism or productivity impairments to caregivers, both of which may present a substantial economic strain. While economic factors are an important element in determining patient access to new treatments, drug approval processes and reimbursement decisions based on Health Technology Assessments, coupled with access to specialist care, strongly influence patient access to current treatments for RA [48].

It is well documented that costs increase with disease duration, severity, and activity of disease, and achieving remission or LDA through early intervention with efficacious therapies could confer notable cost savings and thereby ease the economic burden of RA.

## Discussion

Despite the wide array of available treatments for RA, clinical and patients’ needs remain unmet across key domains such as pain, physical function, mental function, and fatigue, which can all adversely affect social function, sexual function, the ability to work, and overall well-being.

The expansion in the pharmacotherapeutic armamentarium witnessed in the last decade and a half since the advent of the biologic era has hugely improved the achievable outcomes for patients with respect to improvement in symptoms and signs, prevention of structural damage, and preservation of functional status. But despite these advances, significant unmet needs remain as listed above and identified in this review of note, in distinction to past generations of patients with RA where joint deformity and consequent disability were very evident to the treating physician, many areas of contemporary unmet need are of a subjective nature and known only to the patient themselves such as fatigue, pain, and mental function. It is therefore important that the treating physician recognizes this, and having identified the issues that concern an individual patient, address them where possible with both pharmacological and non-pharmacological interventions as appropriate.

It is well documented that patients want to feel engaged and empowered in their treatment, with aspirations that focus on, but are not limited to, reduction in pain, joint swelling, and general well-being. However, reduction in inflammation, prevention of structural damage and incapacity, and achieving remission are regarded by physicians as key treatment goals [31, 49–51]. A recent patient focus group reported that, according to a holistic approach to RA management, pharmacotherapy is an important but not the sole element, in determining clinical outcomes [52]. Indeed, the Assessment of Spondylo Arthritis international Society (ASAS)/European League Against Rheumatism (EULAR) recommendations have a prominent emphasis on body functions and structures, whereas patient-centered care requires non-pharmacological and psychosocial strategies to complement the effects of therapeutic agents [53]. In this regard, the patient focus group emphasized the importance of a multidisciplinary approach that may be instrumental in optimizing the treatment of RA, facilitating improved patient education and dialog between patients and physicians [54].

A recent questionnaire, which included multiple choice, multiple response, and open-ended questions, showed that one-third of respondents reported that they always or usually experienced symptoms of RA that their rheumatologists do not believe or understand, possibly indicative of poor physician–patient communications and/or shortcomings in education. Moreover, the majority of respondents stated that additional medications were needed beyond disease treatment to help control the remaining symptoms of RA, with only 8 % of respondents stating that their symptoms were completely relieved by DMARD or biologic therapy [55].

This literature review revealed that a substantial proportion of patients with RA believe that the core symptoms remain inadequately addressed. The literature consistently supports intensive treatment strategies, with combination DMARDs or combinations of DMARDs and biologics providing more effective control of RA compared with monotherapy or continuation with csDMARDs. These findings were echoed in the economic data, highlighting the overall cost benefits of an effective, intensive therapy. Taken together, data assessed in this review highlight the need for alternative, novel agents that address the multifactorial nature of RA, and ultimately bridge the gap between patients’ and physicians’ treatment goals and aspirations.

## Conclusions

Despite advances in treatment that have helped to improve outcomes for patients with RA, treatment goals, aspirations, and expectations are seldom met for both patients and physicians. RA continues to present a considerable human and economic burden. Novel treatment approaches for RA need to be tested for their ability to ameliorate contemporary unmet need.
